# NiaoDuQing granules relieve chronic kidney disease symptoms by decreasing renal fibrosis and anemia

**DOI:** 10.18632/oncotarget.18473

**Published:** 2017-06-14

**Authors:** Xu Wang, Suyun Yu, Qi Jia, Lichuan Chen, Jinqiu Zhong, Yanhong Pan, Peiliang Shen, Yin Shen, Siliang Wang, Zhonghong Wei, Yuzhu Cao, Yin Lu

**Affiliations:** ^1^ Jiangsu Key Laboratory for Pharmacology and Safety Evaluation of Chinese Materia Medica, School of Pharmacy, Nanjing University of Chinese Medicine, Nanjing, P. R. China; ^2^ Jiangsu Collaborative Innovation Center of Traditional Chinese Medicine Prevention and Treatment of Tumor, Nanjing University of Chinese Medicine, Nanjing, P. R. China

**Keywords:** chronic kidney disease, traditional Chinese medicine, systems pharmacology, fibrosis, anemia, Pathology Section

## Abstract

NiaoDuQing (NDQ) granules, a traditional Chinese medicine, has been clinically used in China for over fourteen years to treat chronic kidney disease (CKD). To elucidate the mechanisms underlying the therapeutic benefits of NDQ, we designed an approach incorporating chemoinformatics, bioinformatics, network biology methods, and cellular and molecular biology experiments. A total of 182 active compounds were identified in NDQ granules, and 397 putative targets associated with different diseases were derived through ADME modelling and target prediction tools. Protein-protein interaction networks of CKD-related and putative NDQ targets were constructed, and 219 candidate targets were identified based on topological features. Pathway enrichment analysis showed that the candidate targets were mostly related to the TGF-β, the p38MAPK, and the erythropoietin (EPO) receptor signaling pathways, which are known contributors to renal fibrosis and/or renal anemia. A rat model of CKD was established to validate the drug-target mechanisms predicted by the systems pharmacology analysis. Experimental results confirmed that NDQ granules exerted therapeutic effects on CKD and its comorbidities, including renal anemia, mainly by modulating the TGF-β and EPO signaling pathways. Thus, the pharmacological actions of NDQ on CKD symptoms correlated well with *in silico* predictions.

## INTRODUCTION

Chronic complex diseases, such as diabetes, chronic kidney disease (CKD), and cardiovascular diseases, seriously threaten the quality of life of millions of people worldwide [1]. It is estimated that up to 10% of the population has various degrees of CKD, and most cases progress to uremia and eventually require kidney transplantation or perpetual dialysis. Therefore, such progression should be prevented [2].

CKD is most commonly a multifactorial clinical syndrome caused by autoimmune reactions, infections, medications, as well as genetic, environmental, and dietary factors, and is associated with tubulointerstitial and glomerular fibrosis [3]. Renal interstitial fibrosis is typified by accumulation of extracellular fibrotic material, infiltration of monocytes and macrophages, fibroblast proliferation/differentiation and tubular atrophy, and involves multiple, inherently complex signaling pathways [4]. At present, there is no cure for this disease, with current treatment strategies mainly relying on blood pressure control through blockade of the renin-angiotensin system. Such approaches only delay the development of end-stage kidney disease and can be associated with serious side effects [5]. Especially, long-term use of these agents could result in therapy resistance and anemia [6, 7]. Thus, due to the lack of therapeutic options, researchers in China and other Asian countries have endeavored to find alternative treatments for CKD and resorted to traditional Chinese herbal drugs, some of which have shown promising results in clinical studies [8, 9].

NiaoDuQing (NDQ) granules, also known as Uremic Clearance Granules, is the first traditional Chinese medicine (TCM) approved by the China Food and Drug Administration to treat CKD (registration information can be accessed here: http://www.sfda.gov.cn/WS01/CL0001/), and has been widely used for over fourteen years in clinical practice [10]. This pharmaceutical composition comprises ten medicinal herbs and has extensive pharmacological effects on CKD, including decreasing serum creatinine (Scr) and blood urea nitrogen (BUN), relieving clinical symptoms of stable CKD patients, delaying renal dialysis, stabilizing renal function and ameliorating renal anemia [11, 12]. On the other hand, many non-SCI indexed Chinese core journals documented the beneficial effects of NDQ for the treatment of CKD, with over 874 cases and no severe adverse reactions reported after an average use of 2.5 months. Therefore, NDQ granules seem especially suitable for long term use.

In most cases, TCMs achieve therapeutic effects by targeting multiple physiological pathways. However, the therapeutically active agents and compounds present on Chinese herbal medicines are numerous, and not easily identified [13]. As the pharmacological mechanism of NDQ and the active substances therein remain as well elusive, developing novel strategies to identify the therapeutic targets and active compounds present in herbal drugs such as NDQ is of great significance.

**Figure 1:**
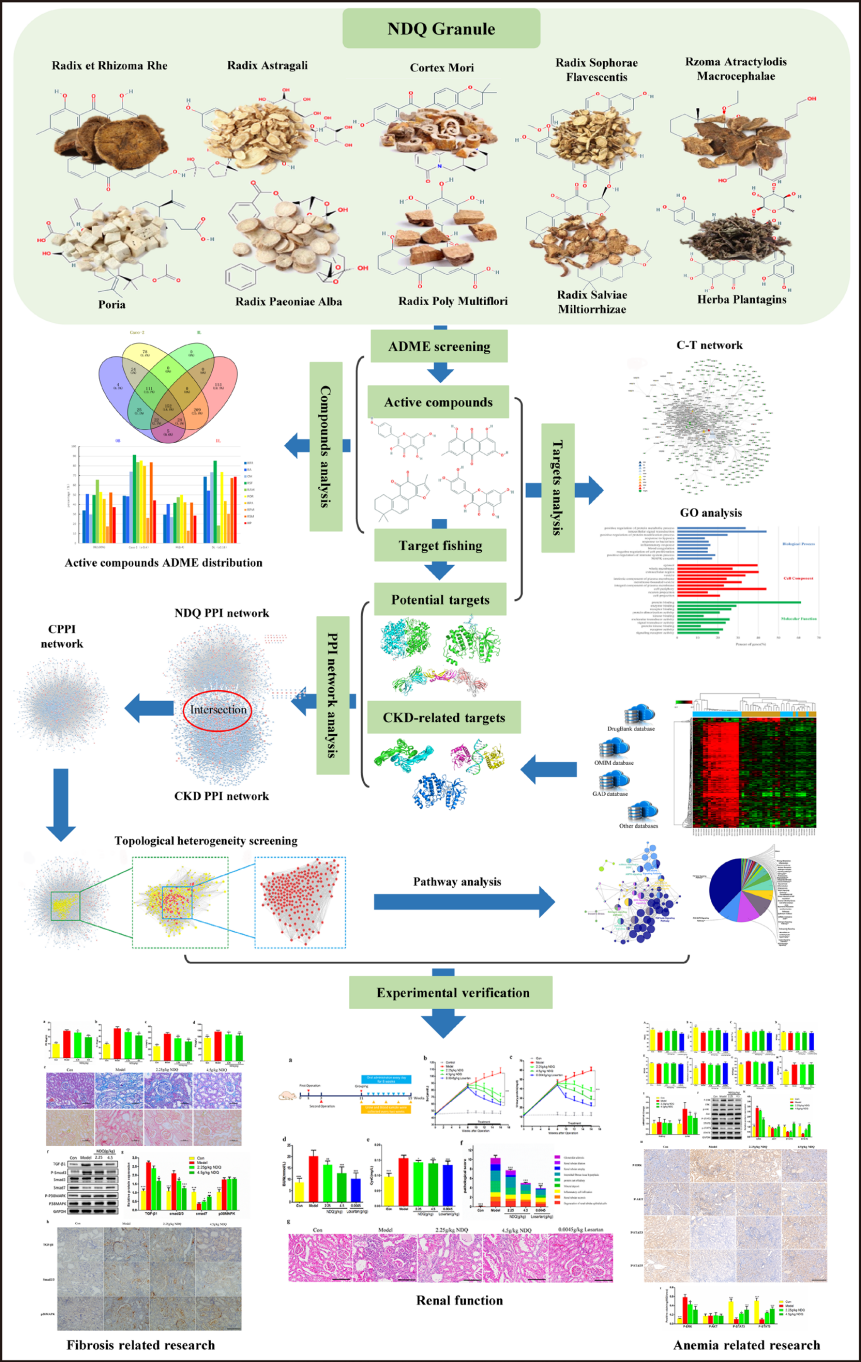
Combined systems pharmacology and experimental approach A combined approach applying systems pharmacology and *in vivo* experiments was developed to identify the active compounds and therapeutic mechanisms of NiaoDuQing (NDQ) granules for the treatment of chronic kidney disease (CKD). The dark blue arrow is the primary line.

As a state-of-the-art technique, systems pharmacology analysis helps to understand the therapeutic targets and active compounds of TCMs by combining drug half-life, oral bioavailability, drug-likeness computations, cell-based intestinal permeability assays, multi-target prediction models, and bioinformatics and network analyses [14–16]. Using a systems pharmacology approach, we herein investigated the pharmacological mechanism of NDQ with the goal of understanding its effects at the system, organ, and molecular levels. Next, we administered NDQ to 5/6 nephrectomy rats and evaluated the ensuing physiopathological and biochemical changes. Importantly, our *in vivo* experimental results largely validated NDQ's mechanism of action, as predicted by the system pharmacology analysis. A flowchart of the study's approach is presented in Figure 1.

## RESULTS AND DISCUSSION

### Screening of NDQ active compounds

Despite considerable efforts and due to their complex chemical compositions, the therapeutic mechanisms of TCMs at the molecular level remain largely unknown. Furthermore, no effective strategies have been specifically developed so far to identify the active compounds in medicinal herbs, which may occur in extremely low amounts and thus escape detection by conventional methods.

A total of 92, 87, 194, 113, 55, 34, 85, 23, 202, and 70 structural compound information results were collected, respectively, for the ten herbs included in the NDQ granules formulation: Radix et Rhizoma Rhei (RRR), Radix Astragali (RA), Cortex Mori (CM), Radix Sophorae Flavescentis (RSF), Rhizoma Atractylodis Macrocephalae (RAM), Poria (POR), Radix Paeoniae Alba (RPA), Radix Poly Multiflori (RPM), Radix Salviae Miltiorrhizae (RSM) and Herba Plantaginis (HP) .

**Figure 2:**
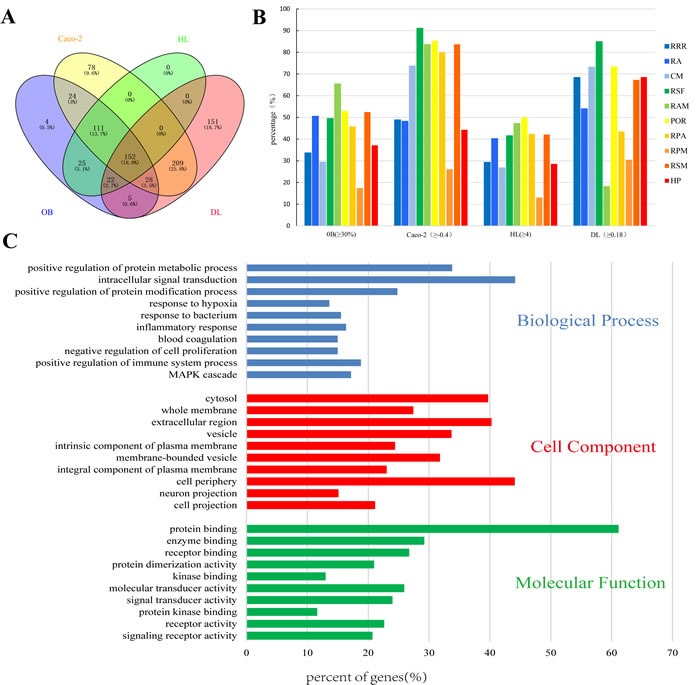
Analysis of the active compounds of NDQ and preliminary GO analysis of putative NDQ targets **A.** Active compounds in NDQ were preliminarily screened for four ADME/Tox parameters. In total, 152 active compounds met the screening criteria. **B.** ADME parameter distribution for different herbs. **C.** GO analysis for Biological processes, Cell component, and Molecular Function terms was performed on putative NDQ targets; the top 10 terms with *P* < 0.05 are shown; Those at the top are the most significant ones.

Active compounds were screened by combining the ADME model with literature confirmation. Four ADME-related parameters for each compound, i.e. drug-likeness (DL), oral bioavailability (OB), Caco-2 permeability (Caco-2), and drug half-life (HL) were assessed based on published findings [15]. The recommended screening criteria were DL ≥ 0.18, OB ≥ 30%, Caco-2 ≥ -0.4 and HL ≥ 4 [17]. A total of 152 potential compounds, including 15 duplicate components, were collected by ADME screening (Figure 2A). As shown in Figure 2B, ∼43% of the molecules (417/955) are orally bioavailable, but only 36.4% (348/955) have long half-life. Interestingly, over 71.4% of the compounds (682/955) were easily absorbed by Caco-2 cell monolayers and approximately 63.9% (611/955) had drug-like characteristics. The numbers of active compounds were 7 for RRR, 14 for RA, 25 for CM, 38 for RSF, 5 for RAM, 14 for POR, 7 for RPA, 1 for RPM, 48 for RSM, and 8 for HP. Additionally, 15 compounds with poor predictive parameters, typical of herbal drugs components, exhibited extensive pharmacological activities against CKD. Accordingly, they were also collected as active compounds for subsequent analyses. In total, 182 compounds with detailed ADME parameter and structural information were collected (Supplementary Table S1). Of the 182 screened compounds, 30 representative ADME-favorable and literature-reported active agents were selected; their ADME parameters and structures are listed in Supplementary Material 1.

### Putative NDQ target prediction and preliminary gene ontology analysis

Complex diseases may sometimes be effectively prevented or treated using TCM formulas, by virtue of the multiple physiopathological processes targeted by the intervening compounds. Thus, we employed a systems target prediction approach to predict NDQ's potential targets on the basis of its active compounds [18]. A total of 413 potential targets were predicted for the 182 candidate compounds: 98 for RRR, 262 for RA, 267 for CM, 211 for RSF, 23 for RAM, 35 for POR, 106 for RPA, 64 for RPM, 189 for RSM, and 153 for HP; of these, 397 targets remained after deleting duplicate ones. Detailed target information is presented in Supplementary Table S2.

A commercial database (QuickGO) [19] based on DAVID for visualization, annotation, and integrated discovery was employed for gene ontology (GO) analysis of the putative NDQ targets described by biological process (BP), cell component (CC), and molecular function (MF) terms. In total, 6461 BPs, 608 CCs, and 1156 MFs that were enriched for this dataset were identified, of which 4791 BPs, 321 CCs, and 717 MFs had *P* values <0.05. Figure 2C exhibits an overview of the GO analysis with 10 remarkably enriched terms in the BP, CC, and MF categories. Terms in the same category are ordered by P values with the cut-off set to 0.05, and those on the top are more significant. The percentage of genes/proteins involved in a term is presented at the bottom of the figure. BP analysis showed that 33.79% of the targets participate in the positive regulation of protein metabolism, while 44.14% are related to intracellular signal transduction. The rest terms Included hypoxic, bacterial, and inflammatory responses, negative regulation of cell proliferation, etc.

CKD is prominently manifested as proteinuria (PRO), which is closely associated with dysfunctional protein metabolism [20]. In parallel, CKD is inevitably accompanied by an inflammatory response, bacterial infection, and mesangial proliferation [21]. Dysregulation of the mitogen-activated protein kinase/extracellular signal-regulated protein kinase (MAPK/ERK) signaling pathway is also known to play a crucial role in renal fibrosis [22]. As revealed by CC analysis, most identified proteins were distributed in the plasma membrane, cytosol, vesicles, or extracellular region. As evidenced by MF analysis, 61.04% and 29.12% of the identified targets could bind proteins and enzymes, respectively. The remaining targets could bind receptors, kinases, etc.

### Compound-target network construction and analysis

**Figure 3:**
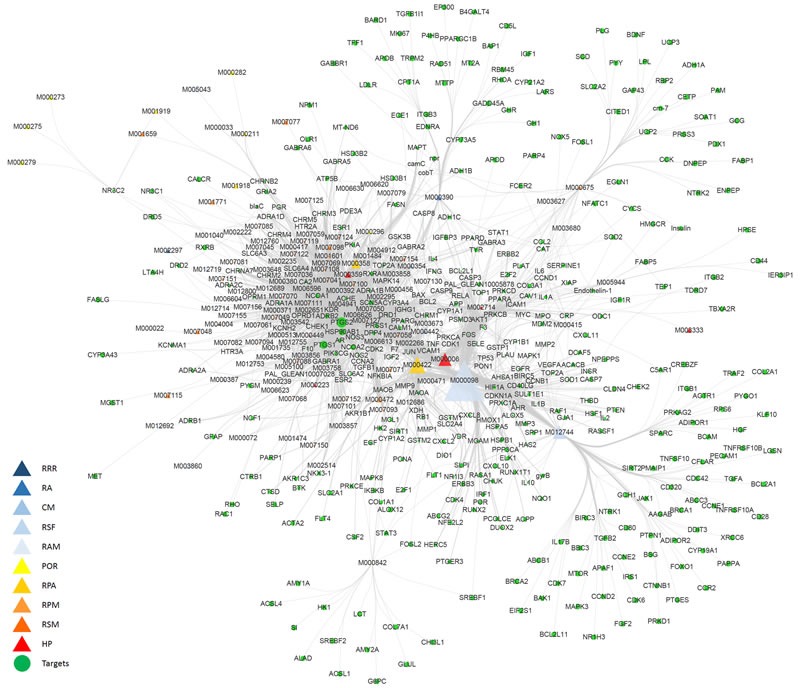
Compound-target network The compound-target network was constructed by linking active compounds (compounds ID) from the different herbs and their potential targets. The nodes represent active compounds (different triangle colors represent different herbs) and targets (green dots). RRR: Radix et Rhizoma Rhei; RA: Radix Astragali; CM: Cortex Mori; RSF: Radix Sophorae Flavescentis; RAM: Rizoma Atractylodis Macrocephalae; POR: Poria; RPA: Radix Paeoniae Alba; RPM: Radix Poly Multiflori; RSM: Radix Salviae Miltiorrhizae; HP: Herba Plantaginis.

By acting on multiple targets, TCM formulas exhibit versatile biological and pharmacological activities. Studying the complicated interactions between TCM compounds and their targets at the systems level may help us comprehensively understand the mechanisms underlying TCMs effects. We constructed a compound-target network based on the candidate NDQ compounds and their potential targets. 571 nodes and 2932 compound-target interactions are embodied in this network (Figure 3 and Supplementary Table S3). The analysis also revealed that quercetin, cinnamic acid, sitosterol, beta-sitosterol, rhein, emodin, hederagenin, mairin, kaempferol, formononetin, caffeic acid, and luteolin are present in multiple herbs, which may have synergistic beneficial effects on patients [23–26] (Table 1). Most of these ingredients have anti-inflammatory actions and renal protective effects. Moreover, luteolin and caffeic acid have well-known antihypertensive potential [24]. Rhein, emodin, and quercetin are the main ingredients of RRR, RA, CM, and RSF, i.e. herbs that have been widely used in China to treat CKD [27]. Cinnamic acid is a compound in RRR and HP that can inhibit platelet aggregation and promote urination [28]. In addition, caffeic acid and beta-sitosterol, which exist in RRR, CM, POR, RSM, and HP, are clinical drugs for CKD adjunctive therapy [29, 30]. As expected, compound-target network analysis revealed a potential synergy between different NDQ components, as they regulate similar processes and targets. For example, oxidative, inflammatory, hypertensive, and fibrotic reactions are commonly counteracted by NDQ through interactions with key genes and proteins such as prostaglandin-endoperoxide synthase 2 (COX-2), transforming growth factor-beta (TGF-β), nuclear factor kappa-B (NF-κB), tumor necrosis factor-alpha (TNF-α), angiotensin I converting enzyme (ACE), etc., which may be beneficial to patients by exerting synergistic effects [31]

**Table 1:** Different herbs contain the same ingredients and have similar effects

Components	Pharmacological activity	Herbs
Cinnamic acid	Hypnagogic, Anti-hypertension, Inhibition of platelet aggregation, Diuresis	RRR,HP
Emodin	Antibacterial, Anti-inflammatory, Immune regulation, Renal protection	RRR,RPM
Rhein	Anti-inflammatory, Antibacterial, Antioxidant, Renal protection	RRR,RPM
Beta-sitosterol	Lowering blood lipid, Anti-inflammatory, Antibacterial	RRR,CM,RPA
Mairin	Anti-inflammatory, Antiviral, Proliferation inhibition	RA,CM,POR
Hederagenin	Antioxidant, Anti-inflammatory	RA,POR
Formononetin	Antioxidant, Proliferation inhibition	RA,RSF
Kaempferol	Anti-inflammatory, Antibacterial, Antioxidant, Anti-hypertension	RA,CM,RPA
Luteolin	Anti-hypertension, Anti-inflammatory, Antioxidant, Antibacterial	RSF,RSM,HP
Sitosterol	Lowering blood lipid, Anti-inflammatory, Antibacterial	RPA,HP
Caffeic acid	Antioxidant, Anti-inflammatory, Anti-hypertension	RSM,HP
Quercetin	Anti-inflammatory, Antioxidant, Inhibition of platelet aggregation, Lowering blood lipid, Antiviral, Anti-hypertension	RA,CM,RSF

### Collection of CKD-related targets

**Figure 4:**
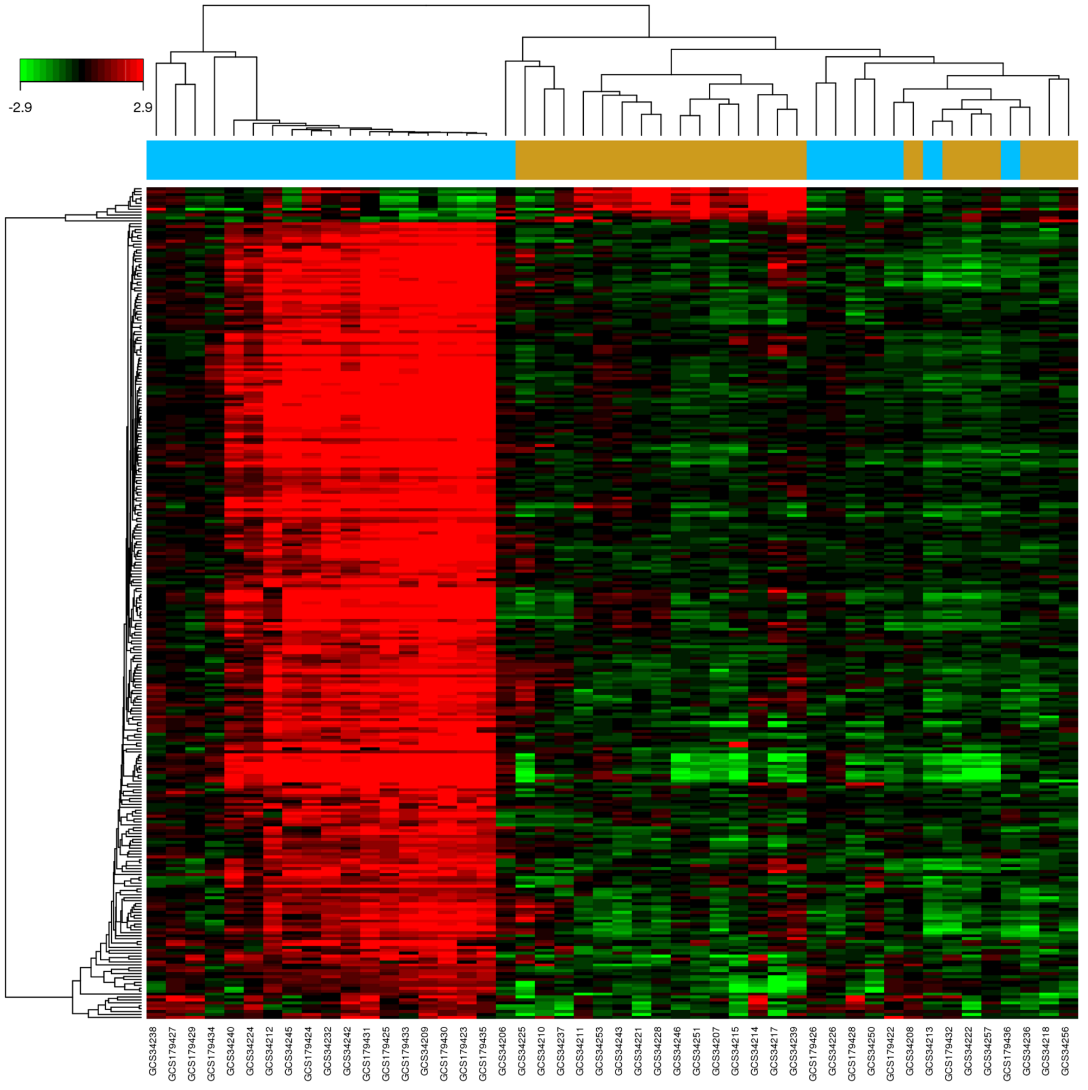
Identification of CKD-related targets by pre-existing microarray data 226 differentially expressed genes identified by the limma package were highly related to kidney disease. Blue and brown stripes represent CKD samples and normal samples, respectively. *P* < 0.01, FC > 2 were considered as cut-off values.

A drug's indication for use is generally determined by the functions of its corresponding targets. We herein collected CKD-related targets from two main sources: differentially expressed genes (DEGs) obtained from publicly available microarray data, and disease-related databases. As shown in Figure 4 and Figure S1, 226 DEGs were identified from the GEO repository microarray data, while other targets were obtained from five databases [32–36]: the Online Mendelian Inheritance in Man (OMIM) database, the DrugBank database, the Pharmacogenomics Knowledge Base (PharmGKB), the Genetic Association Database (GAD), and the Therapeutic Target Database (TTD). After removing redundancy, 513 CKD-related targets were collected (Supplementary Table S4). Of these, 70 were also targets of the herbs comprising NDQ, which suggests an obvious therapeutic potential for this TCM formula.

### Identification of candidate NDQ targets for CKD treatment

Increasing evidence in network biology indicates that genes and proteins do not function independently, but work instead on multiple levels via interconnected molecular networks and pathways [37]. Therefore, we selected proteins as nodes for establishing the network. To clarify the pharmacological mechanism by which NDQ ameliorates CKD, we constructed protein-protein networks that may reflect the behavior and properties of biological molecules [38]. First, a putative target PPI network of NDQ related genes was obtained by screening using the systems pharmacology platform (4589 nodes and 92143 edges) and by database retrieval of the PPI network of CKD-related targets (8253 nodes and 148610 edges; Figures. S2-3). We then merged these two networks to obtain a core protein-protein interaction (CPPI) network that consisted of 3846 nodes and 85232 edges (Figure S4, Table S5).

**Figure 5:**
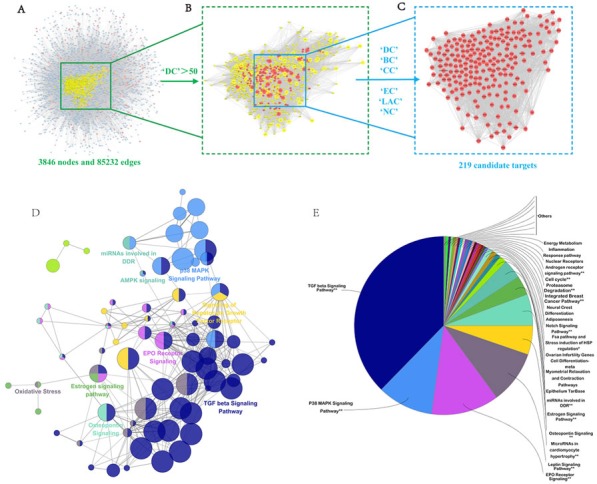
Candidate target identification and ClueGO pathway analysis **A.** Core protein-protein interaction (CPPI) network of NDQ targets. This network consists of 3846 nodes and 85232 edges. **B.** Big hubs of the NDQ CPPI network extracted from (A) whose degree is more than twice the median degree of all nodes in the network. **C.** PPI network of main NDQ targets extracted from (B) by calculating 6 topological features. **D.**-**E.** A functionally grouped network of enriched categories was generated for the target genes. GO terms are represented as nodes, and node size represents the term's enrichment significance. Functionally related groups partially overlap. Only the most significant term in the group was labeled. Representative enriched pathway (*P* < 0.05) interactions among the main NDQ targets.

Subsequently, candidate CKD targets of NDQ were screened by using the topological features of CPPI. According to a previous study, a node was identified as hub if its degree exceeded twice the median degree of all nodes in a network [39]. Using a Cytoscape plugin (CytoNCA), the main hubs of the network were screened by calculating topological features for each hub [40]. The median values of ‘BC’, ‘DC’, ‘EC’, ‘CC’, ‘NC’ and ‘LAC’ were 3095.005112, 83, 0.019282892, 0.475513233, 21.50450205, and 15.77586207, respectively. Thus, 219 hubs with ‘BC’ > 3095.005112, ‘DC’ > 83, ‘EC’ > 0.019282892, ‘CC’ > 0.475513233, ‘NC’ > 21.50450205, and ‘LAC’ > 15.77586207 were set as the main hubs. A flow chart of core targets screening is presented in Figures 5A-5C. Detailed topological features of the CPPI and 219 candidate targets are shown in Supplementary Table S5.

### Pathway enrichment analysis for candidate NDQ targets

A Cytoscape plugin, ClueGO [41], was next applied to further define the pathways involved in the biological networks identified above. As shown in Figures 5D-5E, candidate targets could mainly be assigned to the TGF-β, the p38MAPK, and the erythropoietin (EPO) receptor signaling pathways. Of these, the first two pathways have well-established roles in hepatic, pulmonary, and renal fibrosis [42]. By activating the p38MAPK signaling cascade and inducing the expression of type I collagen (COLI), TGF-β leads to the deposition of collagen and fibronectin [43]. Some typical components and targets of these two pathways, such as Smad2 and Smad3, catenin beta-1 (CTNNB1), SMAD-specific E3 ubiquitin protein ligase 1 (SMURF1), V-akt murine thymoma viral oncogene homolog 1 (AKT1), signal transducer and activator of transcription 3 and 5 (STAT3; STAT5), TNF receptor-associated factor 6 (TRAF6), V-myc avian myelocytomatosis viral oncogene homolog (MYC), mitogen-activated protein kinase 1 (MAPK1), and Jun proto-oncogene (JUN), were also candidate targets of NDQ. As these targets are closely associated with renal fibrosis, it can be inferred that NDQ may exert therapeutic effects by alleviating this symptom. Another NDQ target is the EPO receptor signaling pathway. EPO is a glycosylated hormone secreted by renal interstitial fibroblasts that maintains erythropoietic homeostasis. Inhibition of the EPO receptor signaling pathway results in renal anemia, a common complication of CKD that decreases the patients’ quality of life and is associated with poor disease outcomes [44, 45]. Thus, NDQ's therapeutic effects may also include mitigating renal anemia and its symptoms. Detailed information of target pathways is shown in supplementary Table S6.

### Experimental validation

#### NDQ improves the biochemical index and pathology of CKD

**Figure 6:**
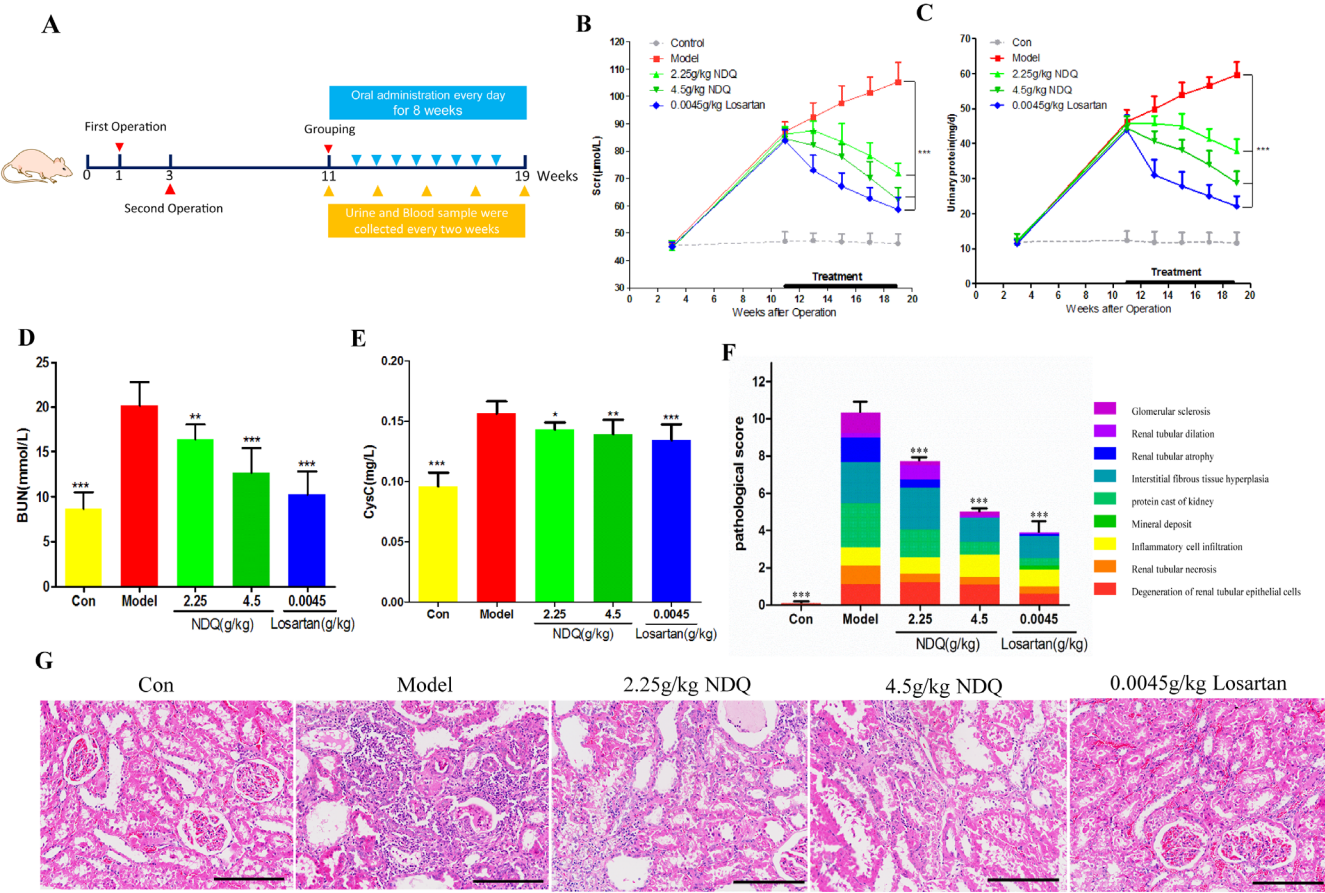
NDQ treatment improves renal function in a CKD animal model **A.** Animal experimental processes. **B.**-**C.** Changes in serum creatinine and urinary protein in 5/6 nephrectomy rats treated for 8 weeks. **D.**-**E.** Serum urea nitrogen and Cystatin C levels in rats at the end of the experiment. **F.** Pathological score of kidney injury. Each color represents a pathological manifestation, the greater the area the more serious the injury. **G.** Representative images of histopathological findings in rat remnant kidneys analyzed on week 19 (HE staining, 200×, scale bar represents 50 μm). Data are presented as the mean ± SD. *p < 0.05, **p < 0.01, ***p < 0.001 (versus untreated model group).

NDQ has been successfully used to treat CKD patients in clinical practice. As mentioned above, we predicted that NDQ was capable of effectively treating CKD by relieving renal fibrosis and anemia. To verify this prediction, we assessed the effects of NDQ on a CKD (i.e. 5/6 nephrectomy) rat model, by assessing comorbidities, kidney function, and histopathology (Figure 6A). Compared with untreated, 5/6 nephrectomized (model) rats, NDQ-treated model animals showed decreased Scr, PRO, BUN, and cystatin C (CysC) during the 8-week treatment (Figures 6B-6E). Meanwhile, a group of model rats treated with losartan, an angiotensin II receptor antagonist used to clinically treat CKD, also showed decreased Scr, PRO, CysC, and BUN. Hematoxylin and eosin staining of rat kidneys showed characteristic pathological lesions including accumulation of extracellular matrix, infiltration of inflammatory cells, and apparent renal interstitial fibrosis and tubular dilation and/or atrophy, where renal fibrosis, as suggested by pathological scores, was a critical part (Figures 6F-6G). In the glomeruli of CKD rats, the mesangial area moderately expanded and the membranes of Bowman's capsules thickened. These pathologic changes were reversed by NDQ and losartan to various degrees, thus ameliorating renal function.

#### Effects of NDQ on fibrosis-related indices and signaling pathways

**Figure 7:**
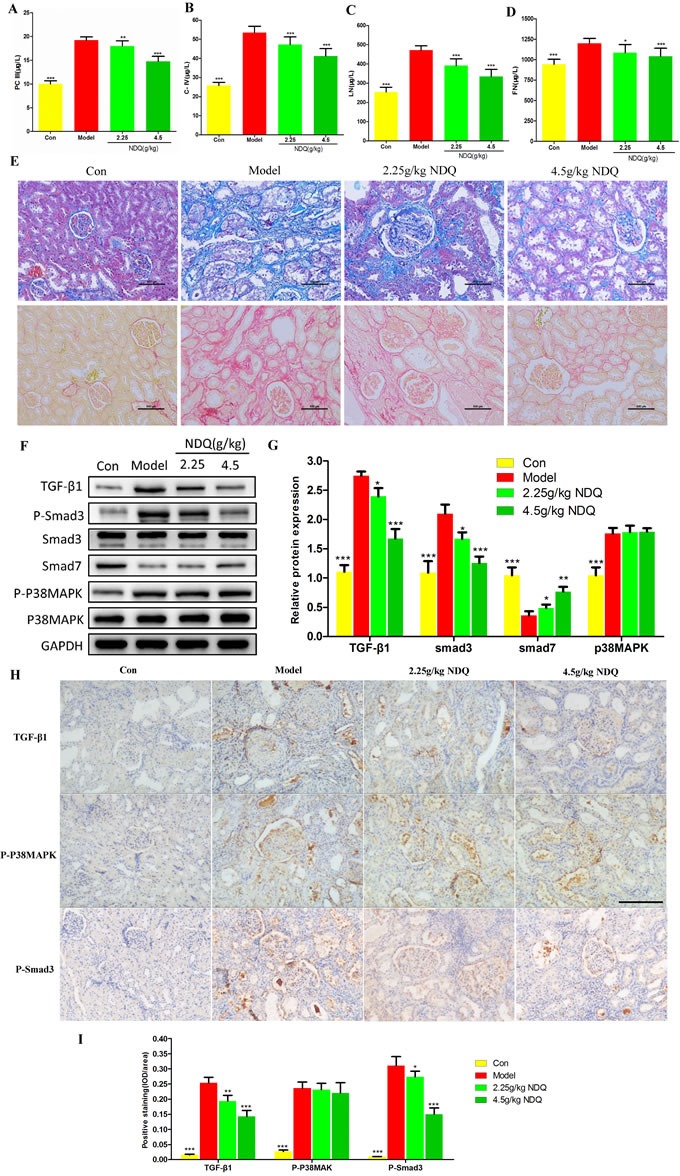
Effects of NDQ on fibrosis-related indices and signaling pathways **A.**-**D.** Four fibrosis-related indexes (PCIII, COLIV, LN, and FN) were measured by ELISA. **E.** Histopathological findings in rat remnant kidneys analyzed on week 19. Renal tissue sections were stained with Masson's trichrome stain for interstitial fibrosis and Sirius red stain for collagen fibers (200×, scale bar represents 500 μm). **F.** Rat kidneys were harvested at the end of the experiment, and analyzed to identify the expression of TGF-β1, Smad2/3, p-Smad2/3, Smad7, p38MAPK, and p-p38MAPK by western blotting. GAPDH served as the loading control. **G.** Densitometric analysis of TGF-β1, Smad2/3, smad7, and p38MAPK expression. **H.** TGF-β1, p38MAPK, and Smad3 expression in the remnant kidney of CKD rats were analyzed by immunohistochemistry (200×, scale bar represents 500 μm). **I.** Expression of TGF-β1, p-p38MAPK, and p-Smad3 was analyzed quantitatively. Data are presented as means ± SD. *p < 0.05, **p < 0.01, ***p < 0.001 (versus untreated model group).

As indicated by the systems pharmacology prediction, a large number of candidate targets of NDQ were related to the TGF-β and p38 MAPK signaling pathways, which are closely related to renal fibrosis [42–45]. By using enzyme-linked immunosorbent assays (ELISA), we first evaluated some classical renal fibrosis-related factors such as type III procollagen peptide (PCIII) [46], type IV collagen (COLIV) [47], laminin (LN) [48], and fibronectin (FN) [49] in 5/6 nephrectomy rats. As shown in Figures 7A-7D, NDQ treatment decreased the serum levels of PCIII, COLIV, LN, and FN. Control CKD rats suffered from severe interstitial fibrosis and extensive infiltration of inflammatory cells, as evidenced by Masson's trichrome and Sirius red staining. Besides alleviating glomerular lesions, NDQ administration also remarkably decelerated the progression of tubulointerstitial lesions. Sirius red staining revealed that the amount of collagen bundles in the kidney decreased in rats treated with NDQ, compared with samples from rats that received normal saline (Figure 7E). Overall, the therapeutic effects of NDQ on renal fibrosis were consistent with those predicted by pathway analysis.

TGF-β receptors transduce signals that regulate the intracellular Smad pathway. Within the latter, Smad2 and Smad3 are particularly involved in renal fibrosis. In renal tubular epithelial cells, the p38MAPK pathway is the main mediator of TGF-β1-induced epithelial-to-mesenchymal transition (EMT) [50]. When activated, this pathway can increase the synthesis of α-smooth muscle cell actin (α-SMA) protein directly, activating the Smad pathway and deposit excess matrix, finally inducing fibrosis [51]. To clarify the mechanism by which NDQ relieved renal fibrosis and to verify the systems pharmacology prediction, we detected TGF-β1, Smad3, phosphorylated Smad3 (p-Smad3), Smad7, p38MAPK, and phosphorylated p38MAPK (p-p38MAPK) by western blotting. They were selected from the candidate targets of systems pharmacology prediction and also played essential roles in the TGF-β and p38MAPK signaling pathways. As shown in Figures 7F-7G, TGF-β1, p-Smad3, and p-p38MAPK expression increased, while the expression of Smad7 was decreased, in the kidneys of untreated CKD rats. All these changes, except for the expression of p-p38MAPK, were significantly reversed by NDQ treatment. Furthermore, an immunohistochemistry assay was conducted to prove the effects of NDQ on fibrosis and related targets. The fibrosis-related proteins TGF-β1, Smad3, and p38MAPK were highly expressed in the untreated model group (Figures 7H-7I), and NDQ administration significantly reversed fibrosis-related indices, without affecting p38MAPK expression patterns. Hence, we deem that NDQ relieves or inhibits renal fibrosis partly by regulating the TGF-β signaling pathway.

#### NDQ attenuates renal anemia by increasing hematological indexes

**Figure 8:**
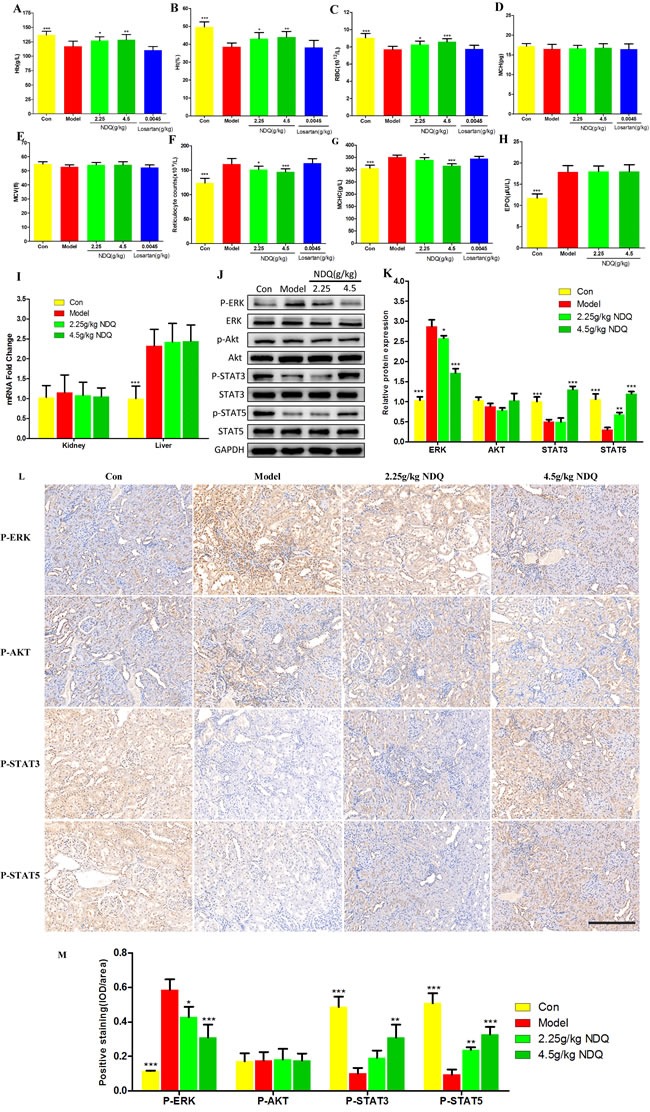
Effect of NDQ on renal anemia and activation of downstream mediators of the EPO receptor **A.**-**G.** Hematological data throughout the study. Hb: hemoglobin; Ht: hematocrit; RBC: red blood cells; MCH: mean cell hemoglobin; MCV: mean cell volume; Rtc: reticulocyte count; MCHC: mean cell hemoglobin concentration. **H.** Serum EPO level was measured by ELISA at the end of the experiment. **I.** EPO mRNA expression in kidney and liver was determined by real-time PCR and normalized to the expression of GAPDH. **J.** Rat kidneys were harvested at the end of the experiment, and tested to determine the expression of p-ERK, ERK, p-AKT, AKT, p-STAT3, STAT3, p-STAT5, and STAT5 by western blotting. GAPDH was used as the loading control. **K.** Densitometric analysis for ERK, AKT, STAT3 and STAT5 expression. **L.** ERK, AKT, STST3 and STAT5 expression in the remnant kidney of CKD rats were analyzed by immunohistochemistry (200×, scale bar represents 500 μm). **M.** Expression of ERK, AKT, STST3 and STAT5 was analyzed quantitatively. Data are presented as means ± SD. *p < 0.05, **p < 0.01, ***p < 0.001 (versus untreated model group).

Pharmacologic treatment of chronic diseases such as CKD is difficult, because single drugs are designed for single diseases in most cases. Nevertheless, a TCM formula developed for a complex disease may also have, unexpectedly, positive effects on other diseases and/or their comorbidities. NDQ therapeutic actions have previously been related to its effects on the EPO signaling pathway, which is highly linked to renal anemia, an early, common CKD complication whose severity increases as renal function declines. Reports indicated that 5/6 nephrectomy rats are prone to renal anemia [45]. Indeed, hemoglobin (Hb) levels, a critical index of anemia [52], were markedly reduced in CKD rats (Figure 8A). Notably, NDQ dose-dependently raised Hb levels, while losartan failed to do so, which is concordant with clinical observations [6]. Furthermore, the hematocrit (Ht) and red blood cell (RBC) count of NDQ-treated rats dropped considerably (*P* < 0.001) compared to those of untreated-CKD rats, although the mean corpuscular hemoglobin (MCH) or mean corpuscular volume (MCV) barely changed. Moreover, CKD rats had significantly elevated reticulocyte count (Rtc) and mean corpuscular hemoglobin concentration (MCHC). All this indicated that CKD rats were anemic. In addition to improving Hb levels, RBC counts, and Ht, NDQ also decreased Rtc and MCHC; losartan, on contrast, hardly caused any improvement, and even deteriorated anemia-associated indices, which is consistent with clinical research [7] (Figures 8B-8G). In this regard, NDQ seems to possess unique advantages over currently used chemical drugs with a single target.

#### Activation of downstream mediators of EPO receptor signaling by NDQ

Renal anemia can mainly be attributed to reduced EPO production from the kidneys or to inactivation of the EPO receptor signaling pathway [53]. To evaluate the impact of NDQ on downstream effectors or the EPO signaling cascade, endogenous serum EPO levels were measured by ELISA in CKD rats at the final stage of the 8-week treatment. EPO levels were significantly increased in 5/6 nephrectomized rats and were not affected by NDQ (Figure 8H). The mechanisms behind such increase were evaluated by measuring EPO mRNA expression in the kidney and liver, i.e. the main EPO-producing organs. Notably, CKD rats showed no significant changes in EPO mRNA expression in the kidney, but liver levels were augmented compared with those of sham rats (Figure 8I). This effect was not altered by NDQ treatment (Figure 8H-8I). Thus, CKD rats showed a marked increase in liver-derived EPO mRNA and protein levels, while NDQ could not counteract this increase.

Since CKD rats are anemic in spite of abnormally elevated EPO levels, we suspected that dysfunction downstream of the EPO receptor signaling pathway and/or decreased reactivity to EPO may underlie this pathological state [54]. Thus, we measured downstream mediators of the EPO receptor signaling pathway, such as phosphatidylinositol 3-kinase/Akt [55], ERK, and members of the receptor-associated Janus family tyrosine kinase (JAK/STAT) signaling pathway, which were also some of the targets predicted by the systems pharmacology approach. Phosphorylation (activation) of ERK [56], enhanced in the kidneys of nephrectomized rats, was attenuated by NDQ. However, Akt activity was affected by neither nephrectomy nor NDQ. On the other hand, STAT3 and STAT5 activation were suppressed in the kidneys of CKD rats, an effect that was dose-dependently reversed by NDQ (Figures 8J-8K). Moreover, an immunohistochemistry assay was conducted to prove the effects of NDQ on anemia and the EPO receptor signaling pathway. The EPO receptor signaling pathway proteins ERK was highly expressed in the untreated model group, while STAT3 and STAT5 expression were decreased, and AKT with no significant changes (Figures 8L-8M). NDQ administration significantly reversed anemia-related indices, without affecting AKT expression patterns. These data suggest that NDQ may relieve anemia through re-activation of downstream mediators of the EPO signaling pathway.

In summary, these data show that NDQ had significant therapeutic benefits on CKD and its comorbidities in a model organism, and provide direct experimental validation of the abovementioned systems pharmacology predictions.

## CONCLUSIONS

Since its approval by China Food and Drug Administration fourteen years ago, NDQ granules have been widely used to treat CKD in clinical practice. Recent clinical data suggested that NDQ delayed the decline in kidney function in a large number of CKD patients, without serious side effects. On the other hand, over 874 cases reported in non-SCI-indexed Chinese core papers ascertained the beneficial effects of NDQ in the treatment of CKD. In this study, the therapeutic targets and active compounds of NDQ were analyzed by using a systems pharmacology approach, as well as compound-target network analysis. Chip analysis was introduced to study the expression of genes highly correlated with CKD. To explore NDQ targets contributing to its therapeutic effects on CKD, a PPI network was constructed and the core targets involved were selected based on topological characteristics. Upon establishing the annotation network, pathways for the potential targets were analyzed by ClueGO, a useful strategy to predict the action mechanism of NDQ.

Subsequently, NDQ was experimentally validated as an effective treatment for CKD, owing mainly to its beneficial effects on renal fibrosis and anemia. NDQ regulated the TGF-β/Smad signaling pathway through suppressing the expression of p-Smad3. Also, Smad7 expression was augmented, which inhibited the activation and function of Smad2 and Smad3. NDQ showed however no significant effects on the p38MAPK signaling pathway, closely associated with EMT in renal fibrosis. In addition, we reported here, for the first time, the mechanism underlying NDQ's beneficial effects on renal anemia, namely the activation of STAT3 and STAT5 downstream of the EPO receptor signaling pathway, which increases EPO-mediated hemostatic balance. Both these proteins were also targets in our systems pharmacology prediction. It is interesting that NDQ can relieve renal fibrosis and renal anemia at the same time, especially since common CKD therapies with drugs like losartan may, after long-term use, aggravate renal anemia.

New prescription rules for TCM herbal formulas used in clinical practice may be uncovered by the findings of this study. Network-based analyses such as those described here may contribute to the holistic understanding and pharmacological evaluation of herbal remedies.

## MATERIALS AND METHODS

### Data preparation

Composite compounds of each herb in NDQ were obtained from TCMSP [15] (http://lsp.nwsuaf.edu.cn/tcmsp.php), at present the largest noncommercial TCM database worldwide. This database is established based on Chinese scientific publications and medical texts, containing over 13731 pure compounds isolated from 505 TCM herbs.

### Screening strategy for active compounds

(1) OB: Oral bioavailability. Representing the percentage of oral administration dose of unchanged drug reaching the systemic circulation, OB reveals the convergence of ADME. Drug-like properties of bioactive molecules, as therapeutic agents, are often determined by high OB.

(2) DL: Drug-likeness. As a qualitative concept utilized in drug design to estimate the ‘drug-like’ degree of a compound, DL helps optimize pharmaceutical and pharmacokinetic properties such as chemical stability and solubility.

(3) Caco-2 permeability. As an effective *in vitro* model, the human intestinal cell line Caco-2 has been used to investigate the passive diffusion of drugs across the intestinal epithelium; the intestinal epithelial permeability is represented by the transport rate (nm/s) of substances through Caco-2 monolayers.

(4) HL: Drug half-life. Drug half-life (t1/2), which is defined as the period of time required for a given amount of a compound to be reduced by half *in vivo*, is the most crucial property because it indicates the timescale over which this compound may work.

Active compounds were selected by setting OB ≥ 30%, DL ≥ 0.18, Caco-2 ≥ -0.4, and HL ≥ 4 as the threshold [57].

### Prediction of drug targets for NDQ

To predict the target profiles of active herbal compounds accurately, an overall drug targeting strategy integrating *in silico* prediction models, chemogenomics methods, and a public database interrogation strategy was performed as previously described [18]:

(1) The *in silico* prediction model efficiently integrates chemical, genomic, and pharmacological information for drug targeting on a large scale, based on two powerful methods: Random Forest (RF) and support vector machines (SVM). In cases where drug targets are identified, proteins with an output expectation value (E-value) SVM > 0.7 or RF > 0.8 are listed as potential targets.

(2) SEA search tool (SEArch, http://sea.bkslab.org/), the online search tool for the Similarity Ensemble Approach, relates proteins based on the chemical similarity of their ligands. The final score is expressed as an E-value indicating the structural similarity of each drug to each target's ligand set.

(3) STITCH 4.0 (Search Tool for Interacting Chemicals, http://stitch.embl.de/), a combined data repository that captures publicly accessible knowledge on chemical-protein interactions derived from experiments, expert-curated databases, and literature by means of text mining.

### Collection of targets related to CKD

Two main methods were used to obtain CKD-related targets. To identify the main DEGs between normal human kidney and CKD specimens, microarray data GSE12682 was downloaded from the Gene Expression Omnibus database (GEO, http://www.ncbi.nlm.nih.gov/geo/). The dataset consists of 52 human samples; after excluding 4 samples that did not pass quality control, 26 normal kidney samples and 22 CKD samples were used to conduct DEGs analysis. DEGs were defined by the Bioconductor/R limma package as previously described [58]. Cut-off values of P < 0.01 and Fold Change |FC| ≥ 2 were applied.

Known targets related to CKD were obtained from five currently available databases using ‘chronic kidney disease’ as the keyword:

(1) DrugBank (http://www.drugbank.ca/, version: 4.3).

(2) OMIM (http://www.omim.org/, last updated: 10th Apr. 2016).

(3) GAD (http://geneticassociationdb.nih.gov/, last updated: 1st Sep. 2014).

(4) TTD (http://database.idrb.cqu.edu.cn/TTD/, last updated: 10th Sep. 2015).

(5) PharmGKB (https://www.pharmgkb.org/index.jsp, last updated: 7th Apr. 2016).

### PPI network construction

PPI data were imported from six currently available PPI databases, including The Biological General Repository for Interaction Datasets (BioGRID), the Biomolecular Interaction Network Database (BIND), the Molecular INTeraction Database (MINT), the Human Protein Reference Database (HPRD), and the Database of Interacting Proteins (DIP), searched by BisoGenet, a Cytoscape plugin [59]. First, an interactive network for the putative NDQ drug targets and known CKD-related targets of NDQ was constructed based on their interaction data. Afterwards, the interaction network was visualized by employing Cytoscape software (Version 3.2.1).

### Definition of topological feature set for the network

By calculating six measures: ‘betweenness centrality (BC)’, ‘degree centrality (DC)’, ‘eigenvector centrality (EC)’, ‘closeness centrality (CC)’, ‘network centrality (NC)’ and ‘local average connectivity (LAC)’ with CytoNCA, the topological properties of every node in the interaction network were analyzed. The definitions and computation equations of these six parameters represent the topological importance of a node in the network. More important nodes receive higher quantitative values within the network [40].

### GO enrichment and pathway analysis

Differentially expressed targets were subjected to GO enrichment using QuickGO to explore their roles in numerous biological processes. A P value cut-off of ≤0.05 was considered significant, and the enriched GO terms were identified with the hypergeometric test. Ten significantly enriched terms in CC, BP, and MF categories are shown. ClueGO, a Cytoscape plugin for visualization of non-redundant biological terms for large gene clusters in a functionally grouped network, was utilized to assess the enrichment of NDQ candidate targets. The ClueGO network was created by using kappa statistics, reflecting the relationships between the terms on the basis of the similarity between their associated genes. The significances of the terms and groups were calculated automatically.

### Animals and drugs

Seventy 8-week-old male Sprague-Dawley rats weighing 180-220g were bought from Shanghai SIPPR/BK Experimental Animal Co. They were raised under controlled humidity (50 ± 10%), temperature (26-28°C), and daily light intensity (12h/12h light/dark cycles), and fed with water and standard diets *ad libitum*. Experimental protocols have been approved by the Committee on Laboratory Animal Care of Nanjing University of Chinese Medicine, and all rats were given humane care according to the guidelines of the National Institutes of Health (USA). NDQ was purchased from KangChen Pharmaceutical Co., Ltd. (20150206). Losartan was obtained from Merck Sharp & Dohme (Australia) Pty. Ltd. (L003135).

### Experimental animal protocols

To establish the remnant kidney model, 5/6 nephrectomy was performed as previously described [60]. Eight weeks after the second surgery, 62 rats remained alive and their urine protein levels and Scr showed significant changes. Based on these parameters, 50 rats were selected and divided into five groups (*n* = 10/group): (I) Sham-operated, receiving orally administered saline daily; (II) CKD control model, given saline every day; (III) NDQ 2.25g/kg (equivalent to the clinical dose), orally administered once a day; (IV) NDQ 4.5g/kg (twice the clinical dose), orally administered once a day; and (V) Losartan 0.0045g/kg (equivalent to the clinical dose for CKD treatment), orally administered daily. Every two weeks, serum was collected to detect urea nitrogen and creatinine levels, and 24h urine was collected from the metabolic cages to measure urine protein levels. In the 16th postoperative week, rats were sacrificed and kidneys and blood were harvested for morphological and biochemical studies. All the procedures were carried out according to the institutional animal care guidelines.

### Biochemical and hematological assays

Blood samples were left to rest for 30 min at room temperature and then centrifuged at 3500 rpm for 10 min at 4°C. The resulting supernatant was aliquoted and stored at 80°C. Scr, PRO, BUN and CysC levels were detected with a Hitachi 7020 Automatic Biochemical Analyzer. Hb, Ht, RBC count, MCH, MCHC, and MCV were determined in whole blood (K3-EDTA) using an automated blood cell counter (HORIBA ABX, Amadora, Portugal). After vital staining with fresh methylene blue (reticulocyte stain; Sigma-Aldrich, USA), Rtc was obtained on blood smears under a microscope.

### Pathological study

Kidney tissues were harvested, fixed in 10% neutral buffered formalin, paraffin-embedded, prepared into 5 μm-thick sections, and stained with hematoxylin and eosin or Masson's trichrome stain using standard methods. Sirius red staining for collagen in stromal structures was performed following an established protocol.

### ELISA

Plasma levels of PCIII, COLIV, LN, FN, and EPO were detected with commercial ELISA kits (R&D Systems, USA). Absorbance was detected at 490 nm with a microplate reader.

### Western blot

Flash-frozen kidney samples were homogenized in whole lysis buffer (250 mmol/L sodium chloride, 10 mmol/L Tris-HCl, 50 mmol/L sodium fluoride, 30 mmol/L sodium pyrophosphate, 10% glycerol, 0.5% Triton X-100, 1 mmol/L phenylmethylsulfonyl fluoride, 1× proteinase inhibitor mixture, 5 mmol/L ZnCl_2_, and 2 mmol/L iodoacetic acid) to prepare whole protein extracts. Protein levels were detected with a Thermo protein assay kit according to the manufacturer's instructions. After separation of total protein (50 μg) with sodium dodecyl sulfate-polyacrylamide gel electrophoresis, the product was transferred by a wet transfer system (Bio-Rad, USA) onto polyvinylidene fluoride membranes. The membranes were thereafter blocked by 5% skimmed milk in TBST buffer [8 g/L NaCl, 2.42 g/L Tris-HCl, and 1 ml/L Tween 20, pH 7.6], incubated with primary antibodies suspended in TBST buffer at 4°C overnight, and then with horseradish peroxidase (HRP)-conjugated secondary antibody. Protein bands were analyzed with a ChemiDoc™ XRS+ system (Bio-Rad, USA).

### Immunohistochemical (IHC) staining

Formalin-fixed, paraffin-embedded kidney tissue samples were cut into 5 μm-thick serial sections for IHC staining. After antigen retrieval using citrate buffer (pH 6.0), the sections were washed with PBS three times, blocked with 10% normal goat serum and incubated with rabbit anti-rat antibodies (Abcam, USA) overnight at 4°C. After three washes with PBS, the sections were incubated with HRP-labeled anti-rabbit IgG antibody (Bioworld, USA) at room temperature for 30 min, washed with PBS and developed with diaminobenzidine. For each section, five middle-power microscopic fields (×400 magnification) were selected randomly to measure staining intensities with IPP software (Image-Pro Plus 6.0, Media, Cybernetics). IOD values were calculated, with the average of the five fields representing the protein expression levels.

### Real-time PCR

Total RNA was extracted from the renal cortex using an RNase mini kit (Transgen, Beijing, China) and reverse transcribed. Primers of EPO were designed and synthesized based on published sequences. Epo (5′-GGGGTGCCCGAACGTC-3′; 5′-GTACCTCTCCAGAACGC-3′; Product length: 120bp). Gapdh (5′-TTCACCACCATGGAGAAGGC-3′; 5′-CTCGTGGTTCACACCCATCA-3′; Product length: 111bp). Real-time PCR was performed using SYBR Green PCR Master Mix (Transgen, Beijing, China) and a 7500 Real-time PCR System (ThermoFisher, New York, USA) according to the manufacturer's protocol.

### Statistical analysis

All data were expressed as percentages and means with standard deviations ($\bar{x}$ ± SD). Statistical analysis and plotting were conducted using Student's *t*-test and one-way ANOVA by GraphPad Prism 5 for Windows. *P* < 0.05 was considered as statistically significant.

## SUPPLEMENTARY FIGURES AND TABLES














