# Supplementary material for: NiaoDuQing granules relieve chronic kidney disease symptoms by decreasing renal fibrosis and anemia
Source: Oncotarget. 2017 Jun 14;8(34):55920–37. doi: 10.18632/oncotarget.18473 (PMC5593534; doi:10.18632/oncotarget.18473)
Supplement: Supplementary file 1 [file oncotarget-08-55920-s001.pdf]

NiaoDuQing granules relieve chronic kidney disease symptoms by decreasing renal fibrosis and anemia

Supplementary Material

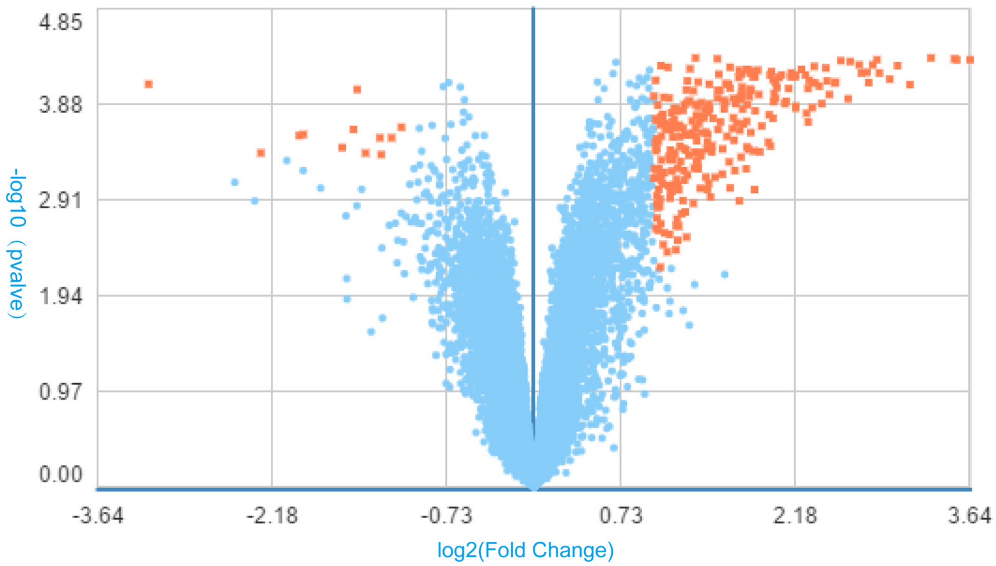

Supplementray Figure 1: Identification of CKD-related targets by pre-existing microarray data. 226 differently expressed genes identified with the limma package were highly related to kidney disease. Volcano plots show differentially expressed genes (red), whereas light blue dots are genes with no significantly different expression. $P < 0.01$ and $|FC| \geq 2$ were the cut-off values.

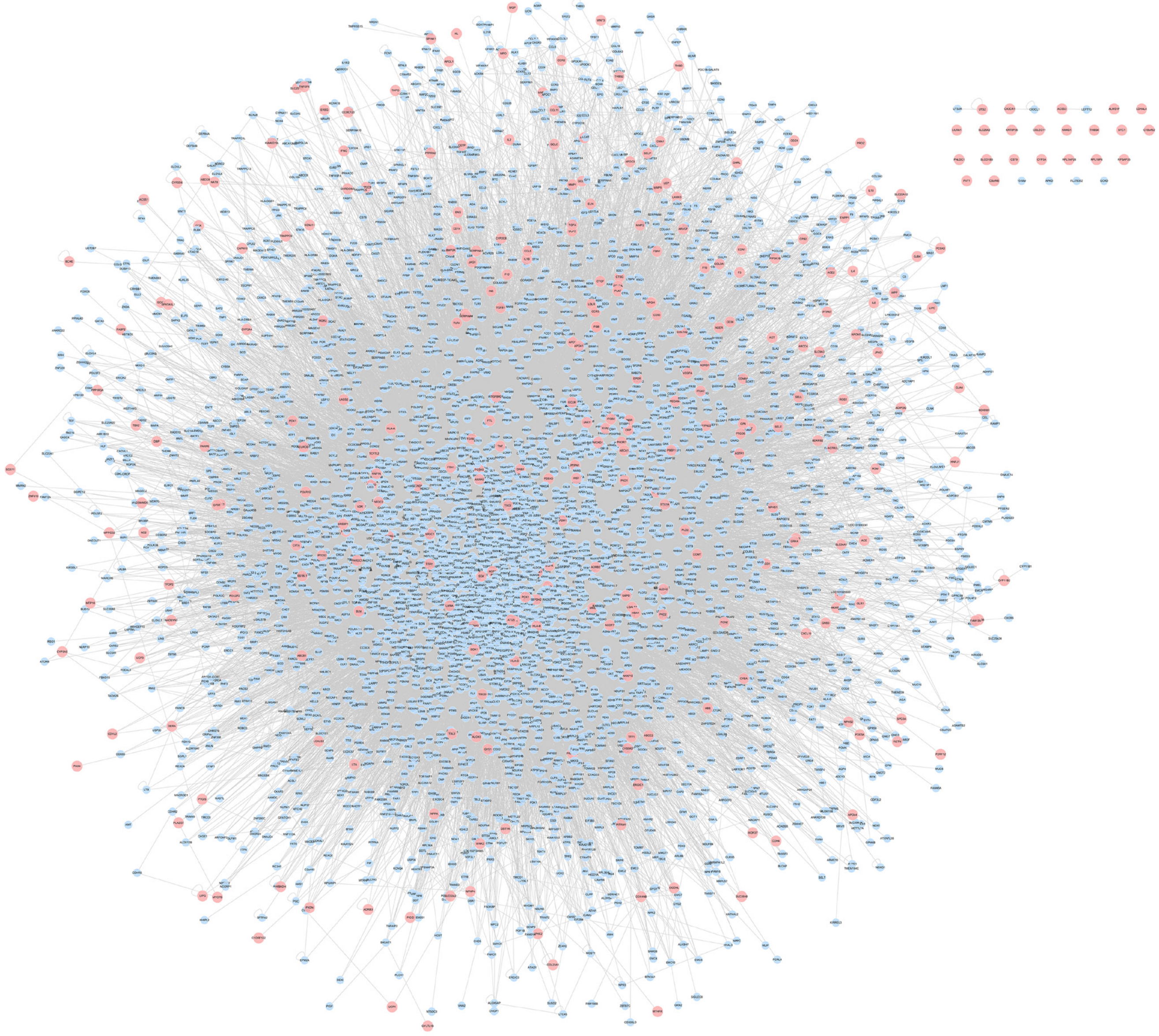

Supplementray Figure 2: PPI network of putative NDQ targets. 4589 nodes and 92143 edges were identified.

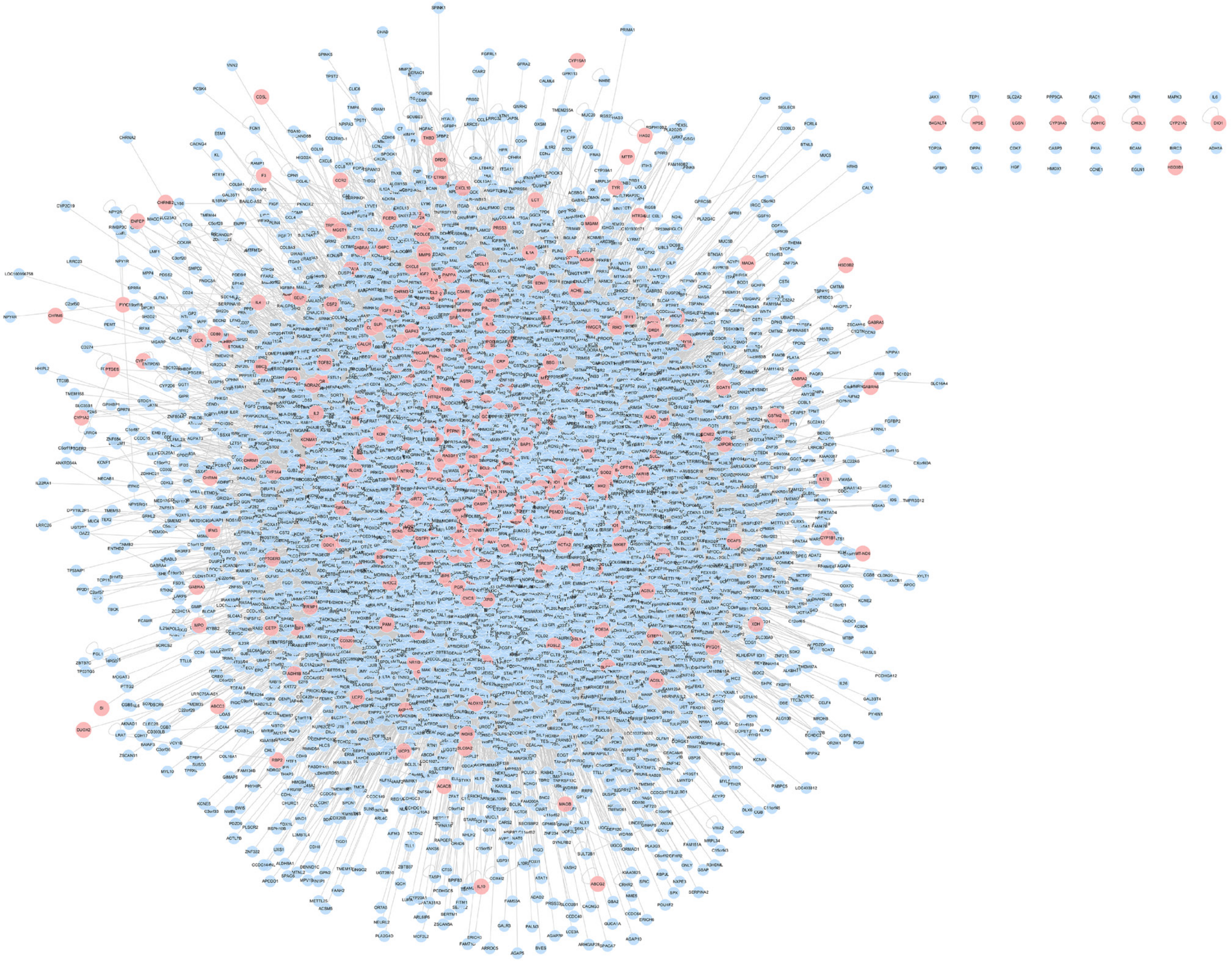

Supplementray Figure 3: PPI network of CKD-related targets. 8253 nodes and 148610 edges were identified.

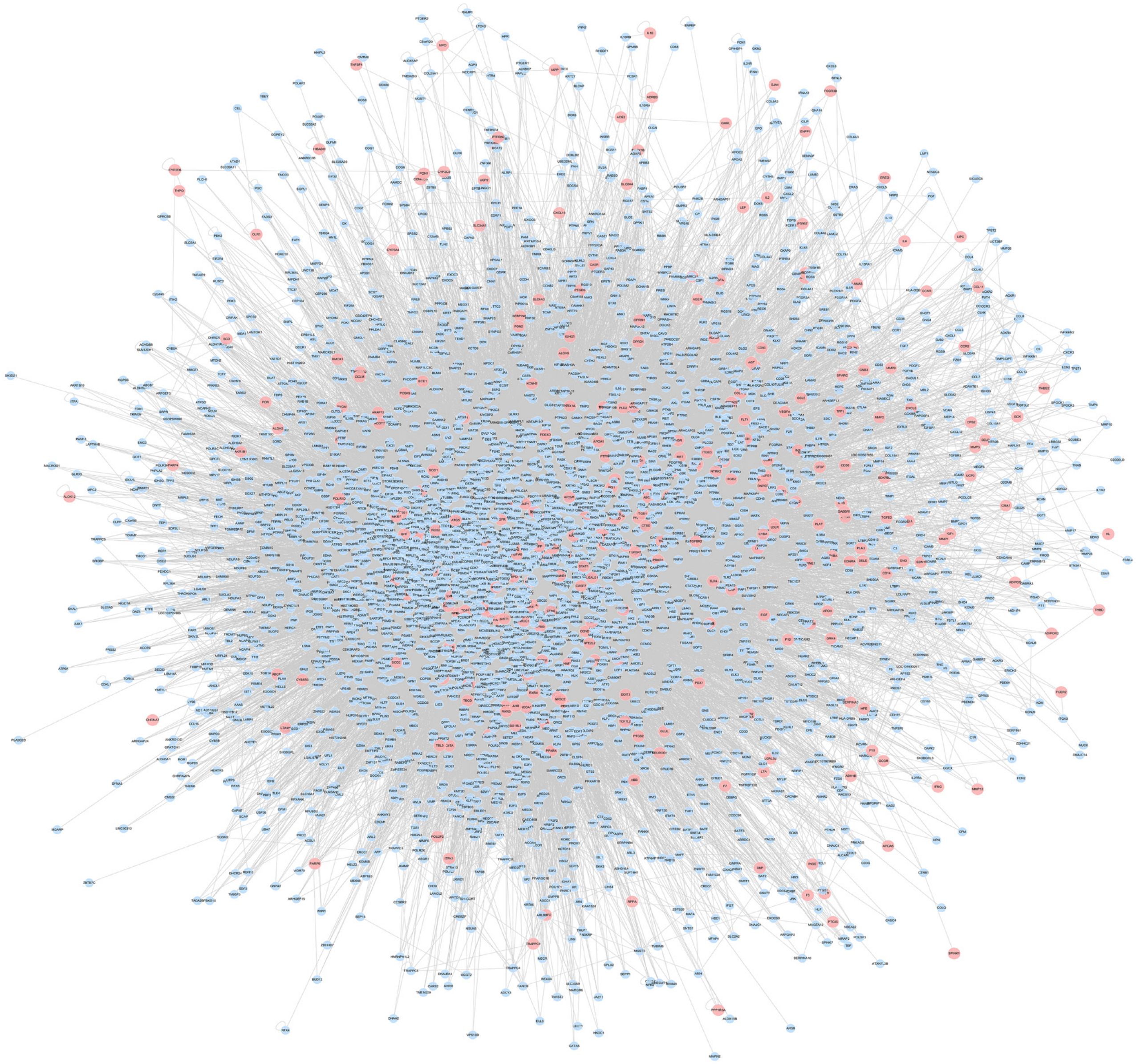

Supplementray Figure 4: Core protein-protein interaction (CPPI) network. The CPPI network consisted of 3846 nodes and 85232 edges.

For Supplementray Tables see in Supplementary Files
